# Food security and the cultural heritage missing link

**DOI:** 10.1016/j.gfs.2022.100660

**Published:** 2022-12

**Authors:** Kofi Britwum, Matty Demont

**Affiliations:** a Department of Agricultural and Environmental Sciences, Tennessee State University, Nashville, TN, USA; b International Rice Research Institute (IRRI), Los Baños, Laguna, Philippines

**Keywords:** Cultural heritage, Biodiversity, Food security, Food systems, Economic resilience, Rice

## Abstract

Though enormous strides have been achieved in recent decades towards reducing food insecurity in the Global South, continued efforts are imperative in light of rapidly expanding populations and threats posed by climate change. A relatively unexplored area in this arena is the nexus between cultural heritage and food security. Cultural heritage embodies indigenous culture, values, and traditions inherited from previous generations. We focus on rice and identify five pathways through which cultural heritage affects food security. Although policy makers face the complex task of balancing trade-offs between preserving cultural heritage and productivity, they can harness cultural heritage to enhance food security by supporting (i) preservation of genetic resources, (ii) valorization, (iii) traditional food processing, (iv) preference matching, and (v) agritourism.

## Highlights

• The nexus between cultural heritage and food security is largely unexplored.
• Indigenous culture, values, and traditions are inherited from previous generations.
• We identify five linkages through which cultural heritage affects food security.
• There are complex trade-offs between preserving cultural heritage and productivity.
• Policy makers can harness cultural heritage to enhance food security.

## 1 Introduction

The success of the Green Revolution and resulting impacts on agricultural production and economic gains still reverberates across parts of Asia, where an explosion of agricultural technologies new to these regions were matched with infrastructural and market developments (Evenson and Gollin, 2003; Hazell, 2009). Despite these successes, eradicating world hunger has proven to be a rather complex, arduous endeavor, intermingled with issues such as poverty (ADB, 2013) and climate change (Wheeler and Von Braun, 2013; Campbell et al., 2016). As a result, substantial pockets of undernutrition remain, e.g., in sub-Saharan Africa, South Asia, Laos, Yemen, Papua New Guinea, and Bolivia (Lenaerts and Demont, 2021). The persistence of hunger in the Global South does not, however, invalidate the successes of the Green Revolution; its programs were not predominantly about eradicating food insecurity (Harwood 2019). As elucidated by Pingali (2012), Green Revolution programs were concentrated in agriculturally suitable regions, suggesting that less favorable areas such as those with poorly developed agricultural systems were largely overlooked. This explains its mixed impact on poverty and food security. When these existing pockets of undernutrition are viewed in the context of other emerging issues such as the anticipated surge in global population over the course of the next 30 years—projected to increase by 2 billion people in 2050—issues of food insecurity become more critical. Indeed, the Covid-19 pandemic which has fractured already tenuous economies in many developing regions has further exposed the disparity between food availability and food access for vulnerable populations. According to preliminary estimates, the pandemic could add anywhere between 83 and 132 million food insecure persons globally (FAO et al., 2020).

Although several initiatives to reduce food production deficits and increase net food supply have yielded positive outcomes, it has been argued that food availability in itself is only one metric within the food security puzzle, and may not be sufficient to turn the tide towards achieving zero hunger. Others have suggested supply chain improvements to harmonize synergies between smallholder producers and suppliers on the one hand, and smallholder producers and consumers on the other (Qureshi et al., 2015). Specific to supply chain bottlenecks which have been escalated by the Covid-19 pandemic, Galanakis (2020) argued that improving transportation infrastructure and reforming trade and taxation policies can facilitate the movement of especially perishable produce from production centers to consumers. Additionally, leveraging technology and advances in communication can help bridge the informational gap between food suppliers and buyers (Galanakis et al., 2021). As the world transitions into a “post-pandemic” era, a focus on sustainability and resilience is vital to build a more robust food system that can withstand future shocks, such as climate shocks (Boyaci Gündüz et al., 2021). Among others, this will require approaches such as a reduction in food waste and an emphasis on food valorization. While these arguments are no doubt compelling, food systems are enmeshed in culture and tradition (Brulotte and Di Giovine 2016; Glover and Stone, 2018; Samaddar et al., 2020; Almansouri et al., 2021; Britwum and Demont 2021a, b; Nichols, 2022). Across many agrarian communities, food production and processing practices and preferences for “authentic” food attributes tend to bear the hallmarks of tradition passed along successive generations. As such, to enhance food security efforts, it is imperative policy is aligned to or leverages food-related cultural heritage. Rahman et al. (2021) echoed similar observations by noting that food cultural heritage and social values are key in driving changes in sustainable agricultural systems, although these have tended to be overlooked.

Cultural heritage embodies indigenous culture, values, and traditions inherited from previous generations. For areas that boast rich cultural heritage, its influence permeates the types of food and how they are produced, value addition, processing, and indigenous preferences for them. Brulotte and Di Giovine (2016), for example, highlighted the relationship between food identity and a people's cultural idiosyncrasies, with documented evidence of such relationships observed across the type of food consumed, mode of consumption, when it is consumed, and with whom it is shared. Strong overlaps have also been reported between heritage food and cultural identity (Rahman et al., 2021), with this relationship dimensioned within the scope of legacy, people, and place (Almansouri et al., 2021).

According to UNESCO, cultural heritage can be considered tangible, such as paintings and archeological sites, or intangible, such as the arts, traditions, and rituals (e.g., see Blake, 2008). Agriculture and food embody aspects of both tangible and intangible cultural heritage, although discussions about food and cultural heritage in the same breath have been relatively nascent. In addition to the inadequate recognition of tangible and intangible aspects of the culture that surrounds local foods, there has been a general lack of inclusion of key stakeholders in the food heritagization processes (Zocchi et al., 2021). In fact, food was first recognized by UNESCO as an intangible cultural heritage as recently as 2010, featuring the Mediterranean diet, the Mexican cuisine, the French Gastronomic meal, and the Croatian gingerbread (UNESCO, 2010). Also noteworthy, framing food within a cultural heritage sphere has wider implications beyond just a cultural identifier. Indeed, food systems are embedded in economic systems which ultimately forge both community and economic identities (Di Giovine and Brulotte, 2016). Daugstad et al. (2006) captured some of these with an observation about shifting paradigms between tangible and intangible cultural heritage, noting that intangible aspects of heritage were being morphed into tangible components, with notable evidence in novel “heritage food” products and opportunities for agritourism. The authors also contended that tangible aspects of cultural heritage had trappings of the intangible, such as having pastures or terraces that provide shared cultural and traditional identities. Similar sentiments were shared by Ramshaw (2016) who asserted that cuisines ascribe a unique heritage value to people groups in specific geographies, from its preparation to consumption. It was noted further that food could serve as a differentiating entity in an increasingly global landscape, leading to the development of a heritage-based identity and a potential touristic attraction.

From the foregoing, it is apparent that identities are established through cultural heritage. Despite this, cultural identities can, and do evolve, and with time, food and commodities not originally native to an area could be adapted to become part of their identity. A case in point is the cocoa crop, which while native to the *Aztecs* and *Mayans* people of South America, have shaped the identities of parts of West Africa since its introduction into these areas in the 19th century (Howes, 1946; Fold, 2001). Other examples include tomatoes, which although originating in western South America (Blanca et al., 2015) has become a fixture in Mediterranean cuisines, or the case of maize which, although originating from central Mexico (Ranum et al., 2014), has become a staple across a wide swath of sub-Saharan Africa.

Apart from food commodities becoming indigenized and coopted into food cultures in regions other than their native origins, there is evidence showing that indigenous technologies are passed down along successive generations. Early Portuguese colonialists to the Upper Guinea Coast in the 16th century, for example, observed a rather sophisticated indigenous rice production system, including the construction of dikes to retain swampy conditions, and transplanting of the sprouted crop on less swampy grounds. The fact that the *Jola* people of Senegal's Casamance region and in Guinea-Bissau and Guinea Conakry used similar techniques to grow rice as recently as the 1960s (Linares, 2002) is testament to the enduring strength of cultural heritage across generations in these areas.

From these discussions, it can be understood that diverse aspects of food production and consumption identities have been, and are shaped by cultural heritage. Not well understood, however, is the nexus between cultural heritage and the “farm to fork” production, supply, and demand cycles, and how leveraging an understanding of this relationship can enhance food security. Incidentally, the literature has been relatively mute about how cultural heritage can be leveraged to enhance and strengthen food security across the Global South. Previous efforts have largely examined cultural heritage within specific contexts such as sustainable ecological systems (e.g., Brabec and Chilton, 2015; Rotherham, 2015; Valkó et al., 2018; Griffiths et al., 2020), agritourism (e.g., LaPan and Barbieri, 2014), commodification and valorization (e.g., Glover and Stone, 2018; Bairagi et al., 2021), diets (e.g., Cuevas et al., 2017; Samaddar et al., 2020), and food preferences (e.g., Demont et al., 2017; Kapelari et al., 2020; Britwum and Demont, 2021a, b; Nichols, 2022).

**Fig. 1:**
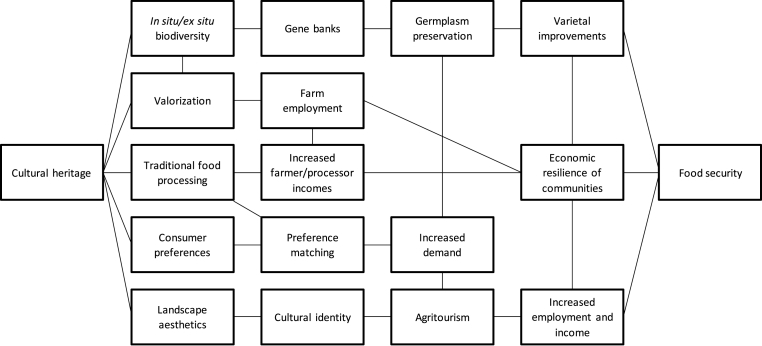
Pathways through which cultural heritage affects food security.

We address this gap by exploring how cultural heritage interacts with food systems and how it can be harnessed to address food insecurity in the Global South. This topic is vast, and the goal of this effort is not to exhaust the plethora of pathways between cultural heritage and food security. Rather, given the broad scope, we delimit the *breadth* of our review to the world's major staple cereal crops, i.e., rice, wheat, and maize, and largely focus on rice, as it has emerged the *de facto* staple of the Global South. Secondly, we delimit the *depth* of our review to five essential aspects through which policy makers can harness cultural heritage to strengthen food systems and food security: (i) preservation of genetic resources, (ii) valorization, (iii) traditional food processing, (iv) preference matching, and (v) agritourism (Fig. 1).

## 2 Cultural heritage, biodiversity, and food security

An important precursor to both food availability and security is genetic diversity, which is closely tied to cultural heritage of historical crop domestication (Fig. 1). In the case of rice, for example, the history of domestication of the Asian rice variety, *Oryza sativa* is well known (Sweeney and McCouch, 2007). What is less understood is that rice is as indigenous to Africa as it is to Asia. The African *Oryza glaberrima* species is believed to have been domesticated about 2000–3000 years ago in the Middle Niger Delta in present-day Mali, from where it spread to two secondary centers of domestication. As mentioned earlier, *Oryza glaberrima* was recorded by early Portuguese colonialists in the region around the 15th and 16th centuries to still be in cultivation by locals along the coast of West Africa (Linares, 2002), described as “the West African rice belt” by Richards (1985), having transcended multiple generations over centuries.

Although *O. glaberrima* has been gradually substituted with the higher yielding Asian variety, *O. sativa*, Linares (2002) reported scattered cultivations of the former in a few West African regions, suggestive of strong cultural ties to ancestors. In addition to cultural heritage, other salient factors have been recognized as contributing to the enduring cultivation of the *O. glaberrima* species across several centuries. Expounding further, Teeken et al. (2012) indicated that the continued cultivation of the African rice variety in different areas of West Africa has been shaped either exclusively or synergistically by natural factors (ecology), cultural factors (having religious and ritual significance), and social factors such as the wars that ravaged parts of the region, where food insecurity, or adaptability to sub-optimal conditions (Mokuwa et al., 2013) encouraged cultivation of local rice varieties adapted to adverse conditions. Even where *O. glaberrima* is still cultivated, there is evidence of variants from interspecific hybridization with *O. sativa* (farmers' hybrid) which have occurred in farmers' fields (Nuijten et al., 2009), with early cultivation traced to Sierra Leone and Guinea Bissau, and subsequently in southern Senegal. Recently though, hybridized varieties such as New Rice for Africa (NERICA) and Advanced Rice varieties for Africa (ARICA) have been bred by scientists, both of which boast superior yields.

With deepening understanding of genetic resources, the significance of genetic diversity within and across regions has become vital. An often-omitted portion of the genetic diversity discussion is the depth of understanding of crop ecology and biodiversity among indigenous people. African rice farmers, for instance, have been adept in managing different varieties often in response to ecological, social, and cultural factors (Teeken et al., 2012). These factors have especially prompted the indigenous development of farmer hybrid rice varieties (between *O. glaberrima* and *O. sativa*) that thrive under poor conditions (Nuijten et al., 2009; Teeken et al., 2012). The original African rice variety remains popular in cultivation in regions that were plagued by wars because of their short duration, or in food insecure areas, given their slow digestibility. This (slow digestibility of African rice) has the advantage of providing a rather lasting satiation after consumption. These observations suggest that while indigenous knowledge partly shaped by cultural heritage has led to the preservation of original varieties, it has also led to the development of local hybrid varieties. In Hindu Kush along the Pakistan-Afghanistan boarder, Abdullah et al. (2021) revealed a wide variety of wild food plants including wild vegetables, fruit, and flavoring spices which are still commonly used by tribal people. These indigenous persons, it was recounted, consumed the wild food plants as food, although the plants also served as markers of cultural heritage. Deb (2014) observed that using certain physical markers and cues such as flowering time, panicle length, grain size and shape, or even color variations, indigenous farmers were able to rid planting fields of species that did not correspond to their needs, maintaining a healthy genetic ecosystem for landraces. While such knowledge steeped in rich cultural heritage has greatly contributed to food production, genetic improvements have played an important role in expanding the frontiers of farming. Recent successes of crop breeding programs have been made possible in part by genetic variability, which is becoming progressively vital as rich genetic diversity strengthens adaptability of plants to diverse environmental pressures, including biotic and abiotic stresses. On the other hand, availability of high yielding crop varieties due to advances in breeding techniques has led to some unintended consequences: genetic erosion and loss of local crop biodiversity (Rerkasem and Rerkasem, 2002).

Defined narrowly, genetic erosion is the loss of genetic diversity of a species, and this has been amplified by recent developments of modern crop varieties—nearly all traditional rice varieties in Asia have been supplanted by high yielding varieties (Kaosa-ard and Rerkasem, 2000; Rerkasem and Rerkasem, 2002). In Bangladesh, for example, some 7000 traditional rice varieties were lost (Hussein, 1994; Thrupp, 2000). Ehrlich et al. (1993) attributed the threat of genetic erosion to (i) massive adoption of select Green Revolution crops at the expense of traditional crop varieties, and (ii) the destruction of natural ecosystems which has had adverse consequences on wild crops. The latter has been pinned to land use changes, industrial-style farming of cash crops, and other large-scale crop production, all of which have facilitated the erosion of local gene pools. Having a restricted diversity of genetic resources, as the trend has unfortunately been, has alarming implications for food security. Some of these concerns were captured by Khoury et al. (2014) in their assessment of global food supplies over the last half century, admitting to a growing homogeneity of global crop supplies and a consequent narrowing of genetic diversity for crops. Thrupp (2000) echoed that these developments could have cascading ramifications across resources such as soils, water, and agrobiodiversity in general, potentially threatening food security. It is also important to stress that genetic erosion has occurred outside of the aforementioned factors. Findings from Mokuwa et al. (2014) revealed morphological differences in *O. glaberrima* species in some parts of West Africa through the interplay of socio-cultural and environmental pressures, all of which have led to restricted genetic diversity of the species. There is also evidence of farmer-instigated rice hybrids between the African and Asian species in farmers' fields predating formal biotechnology in West Africa (Nuijten et al., 2009). Interestingly, some local farmers in Guinea Bissau still claim these “hybrids” as native. These developments underscore the need to preserve local/native species, given the pace of homogenization. Thankfully, the recognition to preserve species has prompted the use of gene banks and habitat conservation of crops.

### 2.1 In situ and ex situ preservation of germplasm

Gene banks and crop conservation programs have yielded promising outcomes, although the influence of cultural heritage in preservation of genetic resources across different regions is not well understood. While loss of biodiversity has been increasingly linked to modern agricultural systems, this relationship need not be adversarial; synergies between modern agriculture and biodiversity can be exploited to roll back losses in the latter. Thrupp (2000) advocated leveraging local knowledge and culture as a means to enhancing and supporting agricultural biodiversity in communities. Towards mitigating the erosion of crop species, we argue that blending agricultural technologies with the culture of communities they are intended to benefit can lead to meaningful gains in production and availability of familiar and native crops. One avenue to redeeming endangered plant varieties is through the development of gene banks. Smale et al. (1998) described crop genetic conservation mechanisms as a pathway to stem the tide of genetic erosion. Specifically, *in situ* and *ex situ* gene banks were mentioned as possible avenues to protect germplasm.

*In situ* and *ex situ* conservation both entail conserving genetic resources and natural habitats. Whereas *ex situ* conservation removes germplasm from their natural evolutionary environments, *in situ* conservation nurtures germplasm within their natural habitats. As narrated by Rerkasem and Rerkasem (2002), initial efforts at preserving germplasm were *ex situ* in the 1970s through several CGIAR national and international gene bank centers. By 2010, there were approximately 1,750 gene banks globally (FAO, 2010). Gene banks are basically large refrigerators that store germplasm, with some popular crops being wheat, rice, and maize. However, storing germplasm *ex situ* means isolating it from its natural, evolutionary ecosystem, which may be detrimental to its continued adaptation (Frankel and Soulé, 1981; Gollin et al., 2000).

With respect to preserving varieties with rich cultural heritage, it has been argued that *in situ* conservation may be a more amenable avenue, as preserving species within their natural habitat also ensures that their population thrives. *In situ* conservation of germplasm can have slightly divergent focal points, one of which is the preservation of a species or an entire ecosystem in protected habitats. Globally, about 70,000 protected areas were estimated in 2007, an uptick from 1997 levels of 56,000 (FAO, 2010). Another focal point of *in situ* conservation is on-farm conservation which entails the preservation of traditional varieties or landraces by rural communities or farmers (Swaminathan, 2002). Suffice to say, *in situ* conservation of germplasm has positive implications towards the functioning of the ecosystem, and across food production and food security. However, *in situ* conservation of varieties may be hampered by climate change as farmers may be reluctant to continue to grow varieties with long growing cycles or low tolerance to dry spells or flooding. It is also worth noting though that farmers' intent of growing traditional varieties may well be nested in cultural heritage and tradition, rather than an interest in conservation (FAO, 2010). It is equally important to highlight that preservation of germplasm in itself may not necessarily be adequate for continued propagation. Indigenous knowledge is critical for the retention of relevant expertise and know-how for continued future cultivation of preserved varieties and species. This makes a strong case for integrating local farmers' knowledge of cultivation methods and managed habitats into genetic/varietal preservation (Nuijten et al., 2009), with such knowledge being vital for cultivation of stored germplasm and consequently, enhanced food security.

### 2.2 Impacts of germplasm conservation on food security

Improving biodiversity and the diversity of genetic resources has discernible impacts across farm systems' resilience, nutrition, enhanced incomes, and food security (Thrupp, 2000). Improved germplasm diversity also boosts farm ecosystems, enhances natural mechanisms for pest and disease control, and improves land fertility. In this context, both *in situ* and *ex situ* germplasm conservation hold enormous promise insofar as trait and agronomic improvements are concerned. Lao People's Democratic Republic (Laos) is a remarkable example of a country that has achieved considerable success in this area, in part the result of its rich cultural heritage in rice. In addition to being a popular staple, rice has enormous religious and cultural relevance in Laos, accompanied by diverse rituals and traditions across different ethnicities (Bestari et al., 2006). Given the cultural and religious constructs around rice, and also the result of centuries-long exchange of seeds among farmers of diverse ethnic backgrounds, Laos boasts one of the highest densities of global rice biodiversity.

Beyond *ex situ* conservation of germplasm, *in situ* conservation has at least an equal stake in meeting food security goals. Indeed, new varieties would have to end up in farmers' fields to have any chance of a lasting impact. Countries such as Ethiopia and the Philippines successfully combined *ex situ* and *in situ* conservation approaches (Jarvis et al., 2010). Ethiopia restored otherwise lost landraces that were previously stored in gene banks and reintroduced them in farmers' fields. In the Philippines, this integrated approach led to the conservation of rice and other crops. Replicating these efforts across other regions and locales thus has the potential to increase productivity and resilience of such communities and consequently, food availability and security. It is crucial for indigenous knowledge of stored germplasm and farmer managed habitats to be integral in these efforts; stored germplasm may not be beneficial if the knowledge to cultivate them, or previously managed environments where they thrived are unavailable.

## 3 Cultural heritage, valorization, and food security

*In situ* conservation may require market incentives that can be generated through “valorization” strategies aiming at giving or adding value to a heritage resource (Fig. 1). Heirloom rice varieties planted by the indigenous peoples of the Philippines' Cordillera Autonomous region hold social, cultural, and spiritual values in addition to a prolonged history of cultivation that spans several centuries (Glover and Stone, 2018). Saddled with production inefficiencies and value chain bottlenecks, Cuevas et al. (2017) and Bairagi et al. (2021) suggested valorizing heirloom rice through place branding, geographic indicators, product differentiation, and promotion of dietary shifts. This, they argued, was salient in light of estimated consumers' willingness to pay (WTP) for “unvalorized” heirloom rice which was observed to be lower than current market prices. From these findings, the association between cultural heritage, valorization, and food security becomes apparent: valorizing heirloom rice could attract a price premium for the varieties, enhance farmers' incomes and possibly production levels, both of which would improve food security in the region (Fig. 1).

Although there is the tendency to view food valorization and value chain improvements through search and experience food attributes, other channels of valorization such as information provision and staging of experiences (Pine and Gilmore, 1999) are equally valid. Bairagi et al. (2021) alluded to this by advocating consumer education regarding the cultural and spiritual significance of heirloom rice, and through the instrumentation of labels that highlight some of these on packaged heirloom rice. Cuevas et al. (2018) went one step further and elicited consumers' value of cultural heritage through their WTP for heirloom rice in the Philippines. The authors staged an experience in which they actively engaged supermarket shoppers in the preservation of the rice terraces and cultural heritage in the Cordillera region through the purchase of heirloom rice products. Despite strong consumer preferences for white rice, the staged experience diverted 15% of shoppers towards pigmented heirloom rice products.

As these findings show, cultural heritage can be leveraged to add value to indigenous food products that have symbolic cultural significance, with positive spillover effects on farmers' incomes and food security. Glover and Stone (2018) described this as “commodification” of the relationship between the history of these varieties and the indigenous communities for which they are native to, in order to draw tangible benefits for them. In summary, valorization of culturally significant crops can lead to improved food security through a combination of “pull” and “push” strategies, i.e., (i) by generating consumer demand for valorized products, and (ii) investing in research and development (seed, agronomy, post-harvest) and infrastructure to increase economies of scale and reduce costs, both of which lead to improvement in farmers' incomes.

## 4 Cultural heritage, traditional food processing, and food security

Integral to the success of food systems and food security efforts are indigenous and modern food processing technologies. Modern processing technologies often alter the product finish, which diverts some of the demand towards products that are minimally processed with traditional technologies and possess the “authentic” product finish. Cultural heritage-induced preferences for the latter attract traditional processors, who increase availability of traditionally processed products on the market. Traditional processing technologies thus contribute to food being available in homes or in the markets in forms traditionally preferred as a result of cultural heritage. They also create a micro-economy, generating income and employment opportunities that strengthen economic resilience of communities (Fig. 1).

Traditional rice in the northern Cordillera Administrative Region in the Philippines makes a fitting case, where it is milled through hand pounding, an activity typically carried out on the farm by women in the rice terrace villages (Glover and Stone, 2018). Today, only a small proportion of heirloom rice is hand pounded, thanks to the introduction of one-pass mills in heirloom rice terraces. Hand pounding results in an imperfectly whitened product finish, which leaves some or all of the pigmented bran on the rice grains rendering the product “authentic.” The case of Chandanpur rice in Odisha, India is similar. Technological upgrading from “traditional” one-pass mills to modern multi-stage mills has been accompanied by quality upgrading of rice. While “modern” rice is well-milled and white, “traditionally” milled rice still features part of the bran and fetches price premiums on the market as it is perceived as authentic and healthier (Custodio et al., 2016). Examined in greater detail, modern versus traditional mills present a conundrum with respect to rice quality which can be viewed from two perspectives: (i) the social construct of quality in the case of modern mills, and (ii) nutritional quality in the case of traditional mills. As such, drawing cultural associations with local rice may still fall short of nutritional quality if local rice is finished through modern rice mills. This calls for a recognition of local rice beyond its cultural markers to include its nutritional benefits (Nichols, 2022), with an emphasis on consuming imperfectly milled rice. In the case of heirloom rice and other local varieties, highlighting nutritional benefits of milled rice that retains the bran can be a selling strategy which can be helpful in the valorization of local rice.

In some cases, compromises are made. Parboiling of rice is traditionally practiced in parts of Africa (Akoa Etoa et al., 2016; Demont et al., 2012) and eastern India as it reduces grain breakage and is perceived as healthier and more satiating, particularly by farmers performing heavy labor throughout the day (Custodio et al., 2019). Parboiled rice, however, features longer cooking time. Due to rising fuel costs and increasing opportunity costs of time, non-parboiled rice and rice with shorter cooking time are becoming increasingly popular. In response to this demand, traditional parboiling processes have evolved to produce “single boiled” rice which takes less time to cook (Custodio et al., 2016). As these examples show, cultural heritage induced preferences for local rice varieties do not only stimulate demand for these varieties, but also revive rice processing technologies and subsequently economic resilience in such communities. While this ultimately contributes to food security goals (Fig. 1), purely milled white rice trails their traditionally milled versions in terms of nutritional quality and calls for nutrition policy that incorporates consumer education on rice quality.

## 5 Cultural heritage, preference matching, and food security

While cultural heritage has inspired food systems across regions both in terms of the types of crops that are indigenous to specific areas and even how they are produced and processed, cultural heritage also defines and structures diets, and hence exerts a significant footprint on food characteristics and attributes that consumers prefer (Fig. 1). It is thus pertinent that these facets are incorporated in the policy discourse on food security. Cultural heritage defines *where* people purchase and consume food (food environments), *when* they consume it (eating occasions), *what* they consume (dishes, food items, beverages), *how* they consume it (using utensils or by hand) and *why* (ingredient attributes and pairings, convenience, consumer attitudes towards food) (Custodio et al., 2021).

It should be acknowledged that cultural heritage does not always lead individuals towards healthier food choices. Gastronomic systems research in eastern India, for example, suggests that culturally inherited preference for starchy and oil dense rice-based diets can be a barrier for nutrition interventions that seek to encourage healthy diets (Custodio et al., 2021; Samaddar et al., 2020). This presents a similar quandary as has been previously discussed, where culturally popular diets are less than nutritious. On the other hand, cultural influences can and have been found to encourage healthier food choices. Recent evidence from India found some consumers preferring bold-grained indigenous “fat rice” over the newly bred improved fine-grained “skinny rice” varieties (Nichols, 2022). Preference for “fat rice” was driven by consumers' experience of them as more satiating than improved rice. Interestingly, the denseness of indigenous rice and the fact that it is eaten with the fingers has led locals to mix it with vegetables (lentils in particular), making the resulting rice-based diets more nutritious than those based on improved “modern” rice.

With the critical role rice plays in assuaging food insecurity, coupled with large foreign exchange reserves expended on imports, many African governments are exploring ways to stimulate local production. Successful substitution of imported with local rice requires that the quality of the latter matches consumer preferences. Consumers in coastal countries featuring major seaports and remote from cultural heritage have been found to develop preferences for the characteristics of imported Asian rice, while consumers exposed to rice cultural heritage either through geographical proximity to loci of African rice domestication or through genealogical ties to the original domesticators have been shown to preserve indigenous preferences for local rice (Demont, 2013; Demont and Ndour, 2015; Demont et al., 2017; Britwum and Demont, 2021a, b). Countries endowed with rice cultural heritage, however, were found to attract lower levels of investment in modern milling (Soullier et al., 2020), presumably because demand for local rice with “authentic finish” is stronger than demand for rice with “import quality finish.” Rice imports have tended to be better milled (more whitened and polished) because the presence of the bran shortens its storability. As such, there is a trade-off between storability and health attributes. Unfortunately, quality perceptions ascribed to better milled white rice is distorting perceptions of quality for local rice, as these perceptions conflate quality with finish rather than with nutrition. Since local brown rice has quite a distinct taste, this will require research in food science and gastronomy (Cuevas et al., 2017) to identify the right dishes and pairings that integrate brown rice into diets.

African policy makers can harness cultural heritage by exploiting its influence over preferred varietal attributes and how this can be integrated with preference matching, processing and genetic resources (Fig. 1) in their National Rice Development Strategies (CARD, 2021). Even crucially, these would have to be woven into nutrition education that highlights the health benefits of local brown rice. For example, the goal of boosting local rice production in the light of a dichotomy of consumer preferences for imported Asian and locally produced African rice spurred breeding priorities towards the development of NERICA varieties, which are hybridized to combine superior resilience and agronomic traits from the heritage African rice variety, *O. glaberrima* and the Asian variety, *O. sativa*. Analyzing results from auction experiments in secondary centers of rice domestication in the Senegambia region, Britwum and Demont (2021a, b) segmented participants based on ethnic affiliation into two groups: those with genealogical ties to original rice domesticators, and those without rice cultural heritage. The authors found that descendants from rice domesticators valued NERICAs more if the products matched the authentic finish of heritage rice, i.e., unbroken grains, while immigrants from the north and northeast without cultural heritage preferred the common “100% broken rice” grade of imports into this region. These results have clear implications for plant breeding and processing. In order to meet food security goals, it is imperative new varieties and food products match preferences in both market segments, inducing cultivation in farmers' fields with cascading positive impacts on food security.

## 6 Cultural heritage, landscape aesthetics, agritourism, and food security

A significant body of literature has examined the economic and social components of traditional agricultural systems. The focus on traditional agricultural systems is typified in parts of the Global South where considerable aesthetic and hedonic values have developed from indigenous farming techniques that boast rich cultural heritage. Farming systems with strong traditional influences retain aspects of both tangible and intangible cultural heritage, the former exemplified by native farmers who have maintained indigenous farming practices, and the latter by individuals outside these locales who tour the sites for their attractions. Examples of these thrive in regions with rice terraces which, while functioning as rice production centers, also attract tourists eager to savor the panorama of these landscapes, and to understand the rich culture that defines them (Tian et al., 2016). Terraces tend to be common in predominantly rural areas, are connected to pre-industrial agriculture, and can be traced to ancient histories (Terkenli et al., 2018). Ultimately, the surge in economic activity has the capacity to boost food security in these and surrounding communities (Fig. 1).

In the Philippines where rice is a national staple, Concepcion et al. (2006) illuminated the multifunctional dimensions of the Ifugao rice terraces, classified by UNESCO as a world heritage site with nearly 2000 years of history; the actual age of the terraces has come under recent scrutiny as new evidence suggest they may only be a couple of centuries old (Acabado et al., 2014). Most importantly, while the symbolism of the terraces is based on a social construction of tradition rather than the divergent viewpoints concerning its ancient origins, terraced rice cultivation in the area is embedded in rich cultural heritage. These span economic, environmental, food security, and cultural impacts (Concepcion et al., 2006). Among the direct impacts of the terraces are economic opportunities through farm incomes and employment opportunities for locals, given that agriculture is a major source of employment in the area. The terraces also serve as an important environmental check with flood control, soil, and biodiversity conservation, in addition to providing food security benefits through domestic rice production and exports to contiguous communities. Cultural identity of terraced farming practices generates revenues for communities through agritourism. It is also easy to recognize that gastronomic experiences of local cuisines are part of the allure that attracts visitors to agricultural heritage sites, with positive implications for local economies (Tian et al., 2016).

It should be noted that the relationship between agricultural terraces, agritourism, economic resilience, and food security (Fig. 1) does not exist by default, suggesting the need for conscientious efforts by relevant stakeholders to nurture these aspects. Terkenli et al. (2018) highlighted supply side interventionist efforts to be crucial, such as infrastructure investment, developing the hospitality industry, and transportation in terraced regions, if the goal is to boost tourism in these areas. These are especially pertinent, given the continued threat of agricultural lands from urbanization. Consequently, some form of geographical seclusion is important for a successful agritourism sector in order to target individuals who value the serenity of agritourism sites outside busy city and urban centers (Kusio and Fiore 2022). Even so, the existence of rich cultural heritage can serve as a buffer in preserving these sites. An ideal example involves the Hani rice terraces in Southwest China with a 1,300-year-old cultural heritage. Although the landscape of agriculture in China is changing, as are other parts of the world due in part to tepid farm incomes and economic opportunities in cities, Hani rice terraces are nearly intact with a devoted preservation by native residents (Zhang et al., 2017). Zhang et al. (2017) observed that the strong bond of culture and heritage surrounding the Hani rice terraces have tended to reinforce notions of preservation by farmers and residents.

Regardless, in order to achieve food security goals, the preservation of terraces tied to cultural heritage need to be assessed against the reality of agricultural productivity from both traditional methods of cultivation and modern agriculture. Ngidlo (2014) examined these issues in four provinces in the Cordillera region in the Philippines. Confronted with low productivity and threatening food security, some farmers in the area transitioned from traditional rice species to modern cultivars since the 1980s. Although the adoption of modern varieties has increased rice production by approximately 80% in the region, the cultivation of these varieties has come at the expense of traditional varieties that boast a wealth of native history. Also worth noting, the expanding needs of local people have prompted the cultivation of non-rice alternatives such as fruit and vegetables, and economic activities such as handicrafts or quarrying (Guimbatan and Baguilat 2006). This could present a policy puzzle between preserving rice terraces in keeping with cultural heritage versus other alternatives that add to food and nutrition security (e.g., dietary diversity) initiatives and expand economic opportunities including modern rice cultivars, fruit and vegetables, and other non-agricultural economic ventures. While there are no easy answers—losing traditional varieties dilutes the authenticity of the history and culture behind terraces which could impact tourism and farm incomes—this consideration is worth noting in any discussion between cultural preservation of terraces and food security. It is instructive to point out that ultimately, traditions are dynamic and undergo transformations from time to time. Consequently, insofar as food security is concerned, some of the newer ventures (e.g., fruit and vegetable cultivation) can be adapted into existing cultural traditions of the terraces. Operationalizing this in practice is understandably nuanced, and would possibly require assessing different regions in light of their unique histories and potential.

Finally, it is worth pointing out that cultural landscapes have been within the threatening orbit of global climate change, which in itself presents another policy dilemma with resource allocation. Climate adaptation and management of heritage sites would thus require a concerted approach such as prioritizing them as environmental resources which need to be managed and protected in a sustainable manner (Aktürk and Dastgerdi 2021).

## 7 Conclusion

Enhancing physical, social, and economic access to safe and nutritious food cannot be achieved without increasing food availability and improving supply chains. However, the fact that significant pockets of food insecurity exist in the Global South despite exponential increase in available food globally in recent decades suggest that more needs to be done beyond these efforts. This review explores five pathways through which cultural heritage can be harnessed to enhance food security: (i) preservation of genetic resources, (ii) valorization, (iii) traditional food processing, (iv) preference matching, and (v) agritourism.

Preservation methods such as *ex situ* conservation and gene banks have played a crucial role in saving germplasm that might otherwise have eroded away. The renewed focus on *in situ* biodiversity can inform breeding priorities in favor of varieties native to particular locales and embedded in rich cultural heritage. The example of Laos can serve as a model for food policy across regions with food cultural heritage. The country has emerged as a hub for rice biodiversity as a result of the synergies between rice cultural heritage and germplasm conservation. Having a rich rice cultural heritage that encouraged seed sharing across ethnicities, and thanks to the establishment of an international gene bank that complemented preservation efforts fostered by cultural heritage, Laos has managed to preserve thousands of rice varieties. We thus argue that a phased introduction of preserved germplasm back in jurisdictions that boast rich cultural heritage can engender farmer participation, improve genetic biodiversity, and ultimately food security. Such an initiative, however, would need to be matched with investments in improvement of soil quality, water availability, and expanded market access.

*In situ* conservation of genetic resources may require valorization to generate the necessary market incentives for farmers to preserve cultural heritage. Policy makers and value chain actors can consider place branding, geographic indicators, product differentiation, promotion of dietary shifts, information provision, and staging of experiences to encourage consumer involvement in the preservation of cultural heritage. Moreover, as lessons from new and emerging urban areas in developing regions have shown, the finish of food is as important as its availability. As such, food valorization through the value chain that incorporates culturally preferred finish and strives to meet consumers' growing expectations of authentic quality can drive local demand, fetch price premiums for farmers, encourage continued cultivation, and forge economic resilience. This would demand supporting investment in traditional food processing technologies that are able to preserve the authentic product finish that connects consumers to cultural heritage and involves them in preserving the latter. In historical centers of rice domestication in particular, food policy makers can leverage the rich cultural heritage in these areas by supporting private sector investment in traditional processing.

Other realms of intangible cultural heritage including the nature of production systems passed along multiple generations have become attraction points for tourists, creating a micro-economy in such regions with positive impacts on incomes and food security. In particular, rice terraces in parts of Southeast Asia with rich cultural heritage have become identity markers for natives who live in these regions and have generated considerable agritourism in these areas. The increased purchasing power from the “tourism economy” can improve economic resilience of indigenous communities and lead to enhanced food security. Policy makers may, however, face complex trade-offs, e.g., in instances where traditional varieties, farming and processing methods embedded in cultural heritage result in low productivity, but where introduction of new varieties, cultivation practices or modern processing attenuates the authenticity of cultural heritage associated with the rice terraces. Policies geared towards agricultural productivity in these regions should thus be cognizant of these trade-offs, and navigate a balance between food availability and the traditions that define such areas.

Finally, it is important to recognize that, cultural heritage, to the extent it builds unhealthy pockets of resistance to more efficient production processes, product quality upgrading, food safety, or healthier diets, could be inimical to food security efforts. This presents another area where policy makers might be conflicted between the preservation of cultural heritage versus the introduction of more efficient production methods or nutritional changes. Rather than disregard or discount the influence of cultural heritage in such instances, nutritionists and policy makers can harness it to improve dietary and food security programs. This will require close collaboration with important custodians of cultural heritage, such as traditional food processors and restaurants, chefs, gastronomy and food scientists, and the tourism industry. Similarly, certain traditional crop varieties may have lower productivity and present a similar “cultural heritage versus high productivity” conundrum. Although policy makers face the complex task of balancing these trade-offs, they can leverage cultural heritage to enhance food security by supporting (i) preservation of genetic resources, (ii) valorization, (iii) traditional food processing, (iv) preference matching, and (v) agritourism. This review is largely exploratory in its attempt at illuminating the role of cultural heritage in food security; further discussions between food security and anthropological constructs are warranted to lend additional insights into this all important but vast topic.

## Funding

We would like to thank all Funders who support this research through their contributions to the CGIAR Trust Fund: https://www.cgiar.org/funders/. In particular, funding from the 10.13039/501100015815CGIAR Initiative on Market Intelligence and the 10.13039/100000865Bill & Melinda Gates Foundation, Seattle, WA, USA [Grant no. OPP1194925] is greatly acknowledged.

## Declaration of competing interest

The authors declare that they have no known competing financial interests or personal relationships that could have appeared to influence the work reported in this paper.

## Data Availability

No data was used for the research described in the article.
